# London

**Published:** 1855

**Authors:** 


### PROGRESS OF EPIDEMICS.

### LONDON.

The following is an abstract of the Epidemics occurring in London during the months of December, 1854, and January and February, 1855, as indicated by the Registrar-General's Report:—

*Scarlet Fever* prevailed extensively in all districts of London, and its weekly mortality was, for the first eight weeks of the period embraced in this report, often more than double of that produced by this disease in the corresponding weeks of the preceding ten years. It was, indeed, equalled in fatality by only the epidemic of 1848-9. During the week ending December 9th, 1854, 100 deaths are recorded as having occurred from this cause; while there were 117 in the corresponding week of 1848. The mortality from this epidemic steadily decreased, till it reached 36 in the week ending March 3rd, 1855.

*Measles.*—The mortality from this disease was generally about the average; it somewhat exceeded that of the preceding ten years, however, in the weeks ending December 16th and January 27th, when the deaths amounted to 49 and 43 respectively. It never approached in intensity the epidemics of some preceding years, as 1845-6 and 1847; in some weeks of which, as many as from 70 to 80 deaths from this disease are recorded.

*Small Pox.*—This disease proved fatal in 36 cases in the week ending December 30th, 1854, and was most prevalent in the north, east, and south districts of London. In the week above referred to, it is mentioned that there were 81 patients in the Small Pox Hospital at Islington, and that the institution was so full that many applications for admission were necessarily refused. The mortality from this disease during each week of the quarter has generally been double of the average of the last ten years, except in the weeks ending January 6th and March 3rd; several years, however, have equalled the present one, and the disease was more fatal during some weeks of the winter 1844-5.

*Hooping Cough* was very prevalent and fatal in London during the winter 1853-4. In the recent season, the mortality from this disease began to exceed the average in the week ending January 6th, and reached the number of 90 in the week ending February 10th; since which time it shewed a slight decline. Speaking generally, the disease appears to have been more fatal than during the corresponding period of any of the preceding ten years.

*Croup.*—The mortality from this disease was often treble of the average; and, in some weeks, was not surpassed in any similar periods of the last ten years. Thus, the deaths from this disease amounted to 17 in the week ending January 13th, and to 20 in the week ending January 20th; the averages for these weeks being respectively 5 and 7. During the last two weeks, the deaths had diminished to 11.

*Influenza* was more prevalent and fatal than usual through great part of this period, but much less severe than the epidemic of 1847-8. The deaths from this disease reached their maximum in the week ending January 27th, when they amounted to 23; after which period they diminished to 5 in the week ending February 24th.

*Erysipelas,* in several weeks, was more fatal than usual.

*Ague,* once very prevalent and fatal, now is only an occasional cause of death in London. In some weeks, 1 or 2 deaths, at most, are recorded; in many weeks none.

*Remittent Fever* proves fatal in 1 or 2 cases weekly; but in some weeks of the period, the mortality from this disease rose to 4 and 5.

*Diarrhœa* varied in intensity, the mortality remaining generally at the average. In the last week, however, it shewed a disposition to increase, the deaths having amounted to 32.

*Dysentery* manifested generally a low rate of mortality; 1, 2, or 3 deaths occurring weekly. The highest number of deaths was 7, in the week ending February 3rd.

*Typhus.*—The mortality from this disease remained nearly at the average throughout the quarter. From 35 to 45 deaths per week generally occurred, with little variation.

*Puerperal Fever* presented no remarkable feature in regard to fatality, except during the first two and last two weeks, when 7, 6, 8, and 7 persons respectively died from it.

*Carbuncle.*—The mortality from this epidemic (which is not in general fatal) was from 1 to 3 per week,—on the whole, somewhat less than that of the preceding two or three years, and shewing a tendency to diminish.

